# Diagnostic Value of Diffusion-Weighted Imaging with Background Body Signal Suppression (DWIBS) for the Pre-Therapeutic Loco-Regional Staging of Cervical Cancer: A Feasibility and Interobserver Reliability Study

**DOI:** 10.3390/curroncol30010089

**Published:** 2023-01-13

**Authors:** Stephan Schleder, Matthias May, Carsten Scholz, Johannes Dinkel, Quirin Strotzer, Ingo Einspieler, Marco Dollinger, Andreas G. Schreyer, Jochen Grassinger, Andreas Schicho

**Affiliations:** 1 Department of Diagnostic and Interventional Radiology, Merciful Brothers Hospital St. Elisabeth, 94315 Straubing, Germany; 2 Department of Urology, Merciful Brothers Hospital St. Elisabeth, 94315 Straubing, Germany; 3 Department of Gynecology and Obstetrics, Merciful Brothers Hospital St. Elisabeth, 94315 Straubing, Germany; 4 Department of Radiology, University Medical Center Regensburg, 93055 Regensburg, Germany; 5 Department of Diagnostic and Interventional Radiology, University Hospital Brandenburg, Brandenburg Medical School Theodor Fontane, 14770 Brandenburg, Germany; 6 Department of Hematology and Oncology, Merciful Brothers Hospital St. Elisabeth, 94315 Straubing, Germany

**Keywords:** MRI, DWIBS, cervical cancer, staging

## Abstract

(1) Background: cervical cancer is one of the leading causes of cancer-related deaths and the fourth most common cancer among women worldwide. Magnetic resonance imaging (MRI) is the modality of choice for loco-regional staging of cervical cancer in the primary diagnostic workup beginning with at least stage IB. (2) Methods: we retrospectively analyzed 16 patients with histopathological proven cervical cancer (FIGO IB1–IVA) for the diagnostic accuracy of standard MRI and standard MRI with diffusion-weighted imaging with background body signal suppression (DWIBS) for the correct pre-therapeutic assessment of the definite FIGO category. (3) Results: In 7 out of 32 readings (22%), DWIBS improved diagnostic accuracy. With DWIBS, four (13%) additional readings were assigned the correct major (I–IV) FIGO stages pre-therapeutically. Interobserver reliability of DWIBS was weakest for parametrial infiltration (k = 0.43; CI-95% 0.00–1.00) and perfect for tumor size <2 cm, infiltration of the vaginal lower third, infiltration of adjacent organs and loco-regional nodal metastases (k = 1.000; CI-95% 1.00–1.00). (4) Conclusions: the pre-therapeutic staging of cervical cancer has a high diagnostic accuracy and interobserver reliability when using standard MRI but can be further optimized with the addition of DWIBS sequences when reporting is performed by an experienced radiologist.

## 1. Introduction

Cervical cancer is one of the leading causes of cancer-related deaths, especially in low- and middle-income countries, and the fourth most common cancer among women worldwide [1,2]. The 2018 FIGO (Fédération Internationale de Gynécologie et d’Obstétrique) system, which is the accepted standard for staging cervical cancer, incorporated results from diagnostic imaging and pathology assessments into stage assignment for the first time [3]. For example, the 2018 FIGO system subdivides stage IB into IB1–3 based on tumor size and assigns lymph node metastases to stage IIIC. Better risk stratification results, using the 2018 FIGO stages, when compared to a prior version of the FIGO classification [1,2]. Parametrial invasion, primary tumor size and loco-regional nodal metastasis predict a poor outcome in cervical cancer [3,4,5,6,7,8,9,10,11]; the five-year survival rate declines from 92% in stage I to 17% in stage IV disease [12].

Magnetic resonance imaging (MRI) is the modality of choice for loco-regional staging of cervical cancer in the primary diagnostic work-up starting with stage IB [13,14]. The superiority of MRI over computed tomography (CT), clinical examination and ultrasound for reliable assessments of tumor size, parametrial invasion, nodal metastases and vaginal extension is well established [15,16,17]. MRI is the diagnostic imaging method of choice, not only for the pre-therapeutic local staging of cervical cancer, but also for the follow-up and evaluation of a treatments’ success [18]. The scan protocol for primary staging includes high-resolution T2-weighted sequences in three planes. Diffusion-weighted sequences can help to assess the exact tumor size and extent, thus minimizing the risk of overstaging, which relies on the differentiation between actual tumor tissue and nearby reactive changes [11]. Moreover, diffusion-weighted sequences play a decisive role in restaging for the evaluation of treatment success or when diagnosing recurrence. The additional use of an intravenously administered gadolinium-based contrast agent is a topic of controversy in the literature without consensus among experts [11]. However, it has been shown that the differentiation between tumor tissue and healthy cervical stroma can be improved by using an intravenous administration of a contrast agent, which particularly benefits the detection of small tumors [19].

However, some drawbacks of MRI in comparison to CT persist, including a longer acquisition time, motion artifacts requiring proficient patient cooperation, a lower spatial resolution and technical challenges related to air–tissue interfaces affecting the image quality of DWI sequences [20].

According to the limitations of traditional DWI imaging methods, Takahara et al. [21,22] developed a novel imaging method, named as “diffusion-weighted imaging with background signal suppression (DWIBS)”, to obtain thin-layer DWI. DWIBS can provide multi-level stimulation and signal average times in a slightly longer time and can effectively inhibit fat by using a short T1 inversion recovery (STIR) echo planar imaging (EPI) sequence, all of which improve the quality of whole-body 3D reconstruction imaging. DWIBS demonstrates a good background signal suppression, which can display lesions and lymph nodes in a stereoscopic and intuitive way. Additionally, DWIBS is less prone to air–tissue border artifacts and signal loss, making it a prime candidate for the diagnostics of near-surface pathologies. Through the black-and-white reversal technology, DWIBS could achieve a similar effect with positron emission tomography (PET) and obtain PET-like images. DWIBS has been widely applied in the clinical diagnosis of brain, nerve and abdomino-pelvic diseases as well as in the staging routine of multiple different cancerous entities [21,22,23,24,25,26,27,28,29]. A larger field of view facilitates the detection of lymph nodes in the pelvic and para-aortic region with an accuracy of 97 and 67%, respectively. A sensitivity of up to 87% and a specificity of 79% in demonstrating vaginal involvement make MRI an important part of preoperative staging of these masses [30].

At present, there is no study evaluating the clinical benefit of DWIBS in the pre-therapeutic staging of patients with cervical cancer, including its diagnostic accuracy and interobserver reliability.

Therefore, the purpose of the presented study was to assess the technical feasibility, interobserver reliability and diagnostic yield of an MRI protocol including contrast enhanced sequences as well as DWIBS in the pre-therapeutic staging of patients with cervical cancer.

## 2. Materials and Methods

### 2.1. Study Design, Subjects and Variables

We identified and retrospectively analyzed 51 female patients with histopathologic proven cervical carcinoma who underwent treatment at our institution between April 2018 and August 2022 and were discussed in our multidisciplinary tumor conference prior to therapy.

For these patients, MRI studies were read independently by two readers who were both blinded to the results of the histopathological report of the definitive surgical resection and for each other’s findings. Reader 1 was a board-certified radiologist with over 11 years of experience in the diagnoses of gynecological cancer and especially in the utilization of DWIBS, whereas Reader 2 was a board-certified radiologist with 3 years of experience in the diagnoses of gynecological cancer without any significant prior experience using DWIBS.

Both readers had to assess the 2018 FIGO cut-off criteria listed in Table 1, first from standard MRI sequences without DWIBS and immediately afterwards with DWIBS, and both readers were asked to assess the rFIGO criteria (radiologic FIGO).

### 2.2. Patient Selection, Inclusion and Exclusion Criteria

Patient inclusion criteria were: (1) histopathological proven cervical carcinoma; (2) preoperative staging with MRI including DWIBS, from which clear images without apparent artifacts were obtained; (3) decision of the multidisciplinary tumor conference for definitive surgical therapy with or without adjuvant chemoradiotherapy.

Patient exclusion criteria were: (1) history of other malignancies; (2) history of neoadjuvant chemoradiotherapy or other treatment for cervical cancer; (3) contraindications for MRI including DWIBS (such as severe claustrophobia and ferromagnetic foreign material).

**Figure 1:**
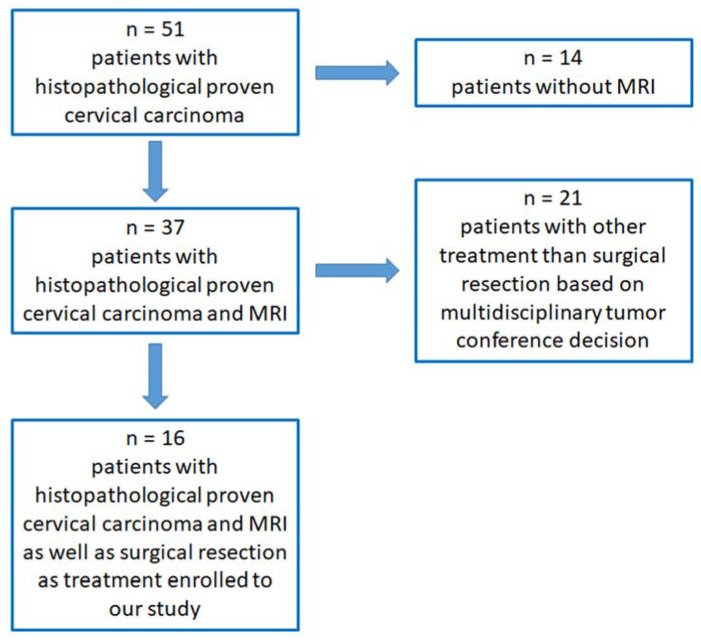
Scheme of enrolled patients for further analysis.

After a thorough review of each single case, 16 female patients were enrolled in our statistical analysis (Figure 1).

### 2.3. MRI Examination

The MRIs were performed using a 1.5 Tesla scanner (Ingenia, Philips Medical Systems DMC GmbH, Hamburg, Germany). The studies were performed with a BODYCOIL system (Philips Medical Systems DMC GmbH, Hamburg, Germany). No bowel preparation was performed before examination. The patients were placed in the supine position and positioned headfirst on the table platform. For the acquisition of the post-contrast studies, an intravenous injection of gadoteric acid (Dotarem, Guerbet Deutschland GmbH, Sulzbach/Taunus, Germany) was performed adapted to each patient’s weight at a ratio of 0.2 mL/kg, varying in a dose of 15–20 mL.

(1) Coronal STIR (short tau inversion recovery; repetition time (TR) 5328, echo time (TE) 50 ms and flip angle (FA) 90°) with a slice thickness of 4 mm, section gap of 1 mm, field of view (FOV) of 300 × 431 × 189 mm, voxel size (VS) 1.00 × 1.48 × 1.00 mm and a scan time of approximately 4 min 26 s.
(2) Sagittal T2-weighted scans without fat saturation (turbo spin echo; TR 3744 ms, TE 90 ms and FA 90°) and with a slice thickness of 4 mm, section gap of 1 mm, FOV of 300 × 300 × 180 mm, VS 0.9 × 1.21 × 4.00 mm and a scan time of approximately 2 min 55 s.
(3) Transversal T1-weighted scans with fat saturation (turbo spin echo spectral pre-saturation with inversion recovery; TR 704 ms, TE 6 ms and FA 90°) with a slice thickness of 4 mm, section gap of 1 mm, FOV of 285 × 461 × 220 mm, VS 0.94 × 1.19 × 4.00 mm and a scan time of approximately 8 min 30 s.
(4) Transversal T2-weighted scans with fat saturation (turbo spin echo spectral attenuated inversion recovery; TR 3744 ms, TE 90 ms and FA 90°) with a slice thickness of 4 mm, section gap of 1 mm, FOV of 255 × 396 × 219 mm, VS 1.14 × 1.44 × 4.00 mm and a scan time of approximately 6 min 55 s.
(5) DWIBS in the axial plane with the following parameters: TR 9795 ms, TE 180 ms, FA 90°, FOV 280 mm × 400 × 276 mm, VS 3.04 × 3.00 × 4.00 mm, slice thickness 4 mm, no section gap, b value 0 and 1000 s/mm^2^ and a scan time of approximately 3 min 25 s.
(6) Contrast enhanced transversal T1-weighted scans with fat saturation (turbo spin echo spectral pre-saturation with inversion recovery; TR 704 ms, TE 6 ms and FA 90°) with a slice thickness of 4 mm, section gap of 1 mm, FOV of 285 × 461 × 220 mm, VS 0.94 × 1.19 × 4.00 mm and a scan time of approximately 8 min 30 s.
(7) Contrast enhanced sagittal T1-weighted scans with fat saturation (turbo spin echo spectral pre-saturation with inversion recovery; TR 692 ms, TE 8 ms and FA 90°) with a slice thickness of 4 mm, section gap of 1 mm, FOV of 289 × 406 × 257 mm, VS 1.13 × 1.36 × 5.00 mm and a scan time of approximately 2 min 30 s.

### 2.4. Statistical Analysis

The standard of reference for the MRI analysis was the histopathological report from the definitive surgical resection.

The statistical analysis was performed using SPSS (version 28, Statistical Package for Social Science, IBM Corp., Armonk, NY, USA). Both readers were tested against each other using standard MRI as well as using MRI with DWIBS. Cohen’s Kappa values were calculated including the 95% confidence interval (95% CI) for the interobserver reliability for both readers. Agreement was interpreted as being poor for κ = 0–0.2, fair for κ = 0.21–0.40, moderate for κ = 0.41–0.60, good for κ = 0.61–0.80 and very good for κ = 0.81–1 [31].

### 2.5. Ethical Considerations

The study was approved by the institutional ethics committee of the Medical Faculty of the University of Regensburg (No. 19-1492-104), and written informed consent was obtained from all patients.

## 3. Results

### 3.1. Diagnostic Efficacy of Standard MRI and MRI + DWIBS

Reader 1 has a better agreement with the histopathologic reference than Reader 2, especially regarding the size of the tumor lesion.

**Figure 2:**
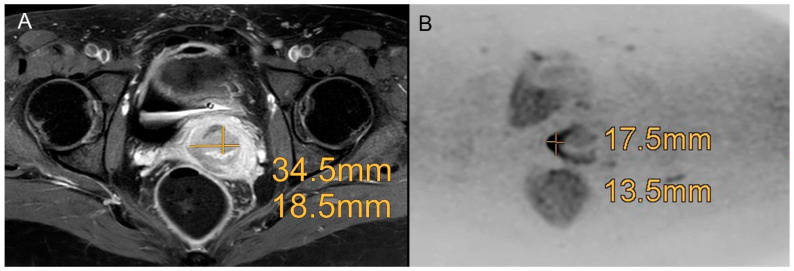
Example of a patient with a FIGO IB1 cervical cancer with a tumor size of 1.9 × 1.5 cm proven by histopathology. Both readers did overestimate the tumor size in the standard MRI ((**A**), contrast enhanced transversal T1-weighted scans with fat saturation) with 3.45 cm × 1.85 cm, and both readers correctly downsized the tumor regarding DWIBS (**B**) with a size of 1.75 × 1.35 cm.

**Figure 3:**
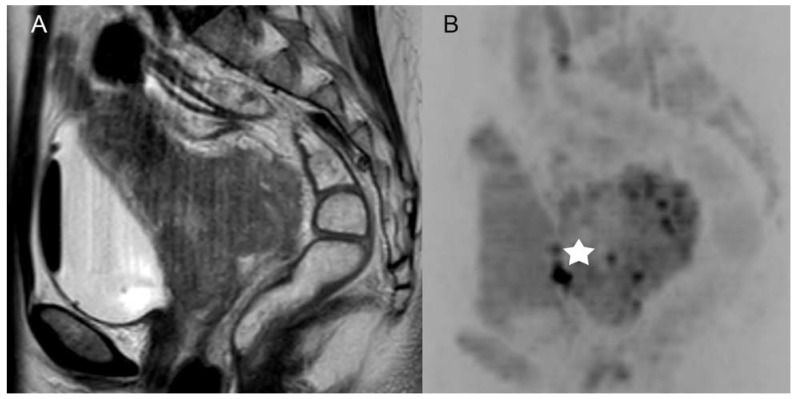
Example of a patient with a FIGO IV cervical carcinoma with a biopsy proven infiltration of the mucosa of the bladder which had not been recognized by both readers in the standard MRI ((**A**), sagittal T2-weighted scans without fat saturation), but was recognized in DWIBS (**B**), marked with a white star (*).

Adding DWIBS, both readers achieved an improvement in diagnostic accuracy of predicting the histopathology verified FIGO stage criteria, particularly regarding the categories “tumor size”, “infiltration of adjacent organs” and “loco-regional LN+”, which is shown in Figure 2 and Figure 3.

In 7 out of 32 readings (22%), additional DWIBS changed the radiologists’ reporting. With DWIBS, four (13%) additional readings were assigned the correct major (I–IV) FIGO stages.

The findings for Reader 1 and 2 compared to the histopathologic standard of reference are presented in Table 2.

### 3.2. Interobserver Reliability Using Standard MRI and MRI + DWIBS

**Figure 4:**
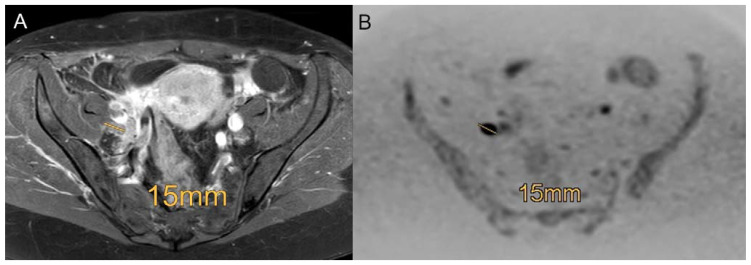
Example of a patient with a FIGO IIIC1 cervical carcinoma with involvement of pelvic lymph nodes using the standard MRI ((**A**)**,** contrast enhanced transversal T1-weighted scans with fat saturation) and DWIBS (**B**) with a right-sided 15 mm lymph node metastasis proven by histopathology. Diagnosis is more reliable in DWIBS (**B**) compared to contrast enhanced transversal T1-weighted scans with fat saturation (**A**).

The agreement between the two readers using standard MRI is good to almost perfect. The only categories without very good interobserver reliability are “TS 2–4 cm” and “TS > 4 cm”, which yield a good interobserver reliability (Table 3). Considering DWIBS in addition to standard MRI, the interobserver agreement becomes more variable regarding the categories of tumor size (“TS 2–4 cm” and “TS > 4 cm”) and the category “parametrial infiltration” with an interobserver reliability of moderate, good and moderate (Table 3). However, all other categories have significantly perfect interobserver agreement (Figure 4).

## 4. Discussion

MRI is the modality of choice for pre-therapeutic staging in women with cervical cancer. Nonetheless, further improvements of diagnostic accuracy seem achievable using new technical approaches, such as whole-body diffusion imaging with background suppression (DWIBS).

The interobserver agreement between the two readers in standard MRI is already substantial to almost perfect. The determination of “tumor size” yields a good interobserver reliability.

Adding DWIBS, both readers achieve an improvement compared with the histopathology reference, particularly regarding the categories “tumor size”, “infiltration of adjacent organs” and “loco-regional LN+”, although the interobserver agreement shows a higher variability with only moderate to good agreement for “parametrial infiltration” and “tumor size”.

In 2021, Pálsdóttir et al. analyzed the interobserver agreement of transvaginal ultrasound and magnetic resonance imaging in local staging of cervical cancer and found the interobserver agreement for the assessment of primary tumor extension in patients with cervical cancer to be moderate for transvaginal ultrasound and moderate-to-good for MRI. DWI sequences–such as DWIBS–were not evaluated [32]. The authors mention, as an unexpected finding, the similarity in interobserver agreement between experienced and less experienced observers, both for transvaginal ultrasound and for MRI with an exception for parametrial invasion (with experienced transvaginal ultrasound observers showing significantly better agreement than less experienced ones) [15,32,33].

Data from a systematic review from 2013 show that MRI (pooled sensitivity 84%; 95% CI 76–90%), especially in the detection of parametrial infiltration and cervical carcinoma >stage IIB, is superior to clinical examination (pooled sensitivity 40%; 95% CI 25–58%), underlining the necessity of pre-therapeutic staging with MRI as the imaging technique [33,34].

Regarding lymph node detection, a meta-analysis showed that MRI with diffusion imaging had the best sensitivity at 88%, PET or PET/CT had the best specificity at 94% and the AUC of DWI and PET/CT was just above 90% in each case compared to the histopathology pointing out the need for diffusion-weighted imaging such as DWIBS [32,33]

The achievable detection rate for transvaginal ultrasound for a tumor extension >4 cm is 78% with a specificity of 99%; for deep stromal infiltration (> 2/3 of the wall thickness) transvaginal ultrasound has a sensitivity of 88–91% with a specificity of 93–97%; and parametrial infiltration has a sensitivity of 60–83% with a specificity of 89–100%). In specialized centers, diagnostically comparable results can be achieved with MRI [33,34,35,36].

Concerning strengths and limitations, the well circumscribed and clearly defined study population with histopathological proven cervical carcinoma who had undergone preoperative staging with MRI including DWIBS without history of other malignancies or prior treatments for cervical cancer and the decision of the multidisciplinary tumor conference for definitive surgical therapy with the histopathological report of the definitive surgical resection as standard of reference for the MRI analysis must be mentioned as a clear strength of the study conducted. On the other hand, the rigorous homogeneity limited our population in this single-center proof-of-concept study to 16 patients. Furthermore, standard diffusion-weighted imaging (DWI) sequences are well established and recommended in MRI protocols addressing cervical cancer but have not been compared to DWIBS in this study. Moreover, with only 16 patients included, a statistically valid comparison between standard MRI and MRI + DWIBS was not feasible and not the primary intention of this study. Those issues will be addressed in further follow-up studies.

Further multi-site and multi-scanner studies need to be set up as our data suggest a relevant clinical benefit for both experienced and novice DWIBS readers. As MRI is already the standard imaging method for loco-regional staging in cervical cancer, the return on investment for the acquisition of one additional non-contrast sequence urges a change in our standard of care. Easily improving diagnostic accuracy at almost no cost is an opportunity which seldom occurs. Early reliable diagnosis and correct rFIGO classification combined with the definite histopathologic results enable a faster and more accurate individual treatment initiation, which greatly benefits clinical outcome.

## 5. Conclusions

The pre-therapeutic staging of cervical cancer using MRI has a high interobserver reliability and accuracy, which is further improved by the addition of DWIBS sequences for both experienced and novice radiologists. Larger multicentric studies should further elaborate on our findings and compare various diffusion-weighted imaging methods.

## Figures and Tables

**Table 1:** FIGO 2018-based categories analyzed by both readers with abbreviations used.

Abbreviation Used	Histopathological Category Analyzed by Both Readers	Associated FIGO ^3^ 2018 Category
TS ^1^ < 2 cm	invasive carcinoma ≤2 cm in greatest dimension (tumor size)	at least stage IB1
TS ^1^ 2–4 cm	invasive carcinoma >2 cm and ≤4 cm in greatest dimension (tumor size)	at least stage IB2
TS ^1^ > 4 cm	invasive carcinoma >4 cm in greatest dimension (tumor size)	at least stage IB3
parametrial infiltration	with parametrial involvement but not up to the pelvic wall	at least stage IIB
infiltration of vaginal lower third	the carcinoma involves the lower third of the vagina	at least stage III
infiltration of adjacent organs	the carcinoma has extended beyond the true pelvis or has involved (biopsy proven) the mucosa of the bladder or rectum.	stage IV
loco-regional LN ^2^+	involvement of pelvic and/or para-aortic lymph nodes	at least stage IIIC1

^1^ tumor size; ^2^ lymph node positivity; ^3^ Fédération Internationale de Gynécologie et d’Obstétrique.

**Table 2:** Histopathological FIGO for each patient included in our study in comparison to the rFIGO for Reader 1 and Reader 2 for standard MRI and MRI + DWIBS, respectively.

Patient Number	Age	TNM ^1^	Histol. FIGO ^4^	MRI ^2^ Reader 1-rFIGO ^5^	MRI ^2^ + DWIBS ^3^ Reader 1–rFIGO ^5^	MRI ^2^ Reader 2–rFIGO ^5^	MRI^2^ + DWIBS ^3^ Reader 2–rFIGO ^5^
1	44	pT2a2 pN1 cM0	IIIC1	IIIC1	IIIC1	IIIC1	IIIC1
2	57	pT1b1 pN1 cM0	IIIC1	IIIC1	IIIC1	IIIC1	IIIC1
3	59	pT2a pN0 cM0	IIA2	IIA2	IIA2	IIA2	IIA2
4	33	pT2b pN0 cM0	IIB	IIIA	IIA2	IIIA	IIA2
5	37	pT2b pN0 cM0	IIB	IIA2	IIA2	IIIA	IIA2
6	43	pT1b1 pN0 cM0	IB1	IB1	IB1	IB1	IB1
7	57	pT4 pN1 cM0	IVA	IVA	IVA	IVA	IVA
8	62	pT2a1 pN0 cM0	IIA1	IIA1	IIA1	IIA2	IIA2
9	42	pT1b1 pN1 cM0	IB1	IB1	IB1	IB1	IB1
10	60	pT1b1 pN0 cM0	IB1	no tumor	no tumor	no tumor	no tumor
11	34	pT1b2 pN0 cM0	IB2	IB2	IB2	IB2	IB3
12	46	pT2b pN0 cM0	IIIA	IIIA	IIIA	IIA2	IIIA
13	37	pT1b1 pN0 cM0	IB1	IB2	IB1	IB2	IB1
14	37	pT4 pN1 cM0	IVA	IVA	IVA	IVA	IVA
15	42	pT1b1 pN0 cM0	IB1	no tumor	no tumor	no tumor	no tumor
16	72	pT1b1 pN0 cM0	IB1	IB1	IB1	IB1	IB1

^1^ TNM classification of malignant tumors; ^2^ magnetic resonance imaging; ^3^ diffusion-weighted imaging with background saturation; ^4^ Fédération Internationale de Gynécologie et d’Obstétrique; ^5^ radiologic rFIGO classification.

**Table 3:** Interobserver reliability for both readers in the evaluation using standard MRI and MRI with DWIBS.

Histopathological Category	Cohen´s Kappa (95%-CI ^1^) for Standard MRI ^2^	Cohen´s Kappa (95%-CI ^1^) for MRI ^2^ with DWIBS ^3^
TS ^4^ < 2 cm	k = 1.00 (1.00–1.00)	k = 1.00 (1.00–1.00)
TS ^4^ 2–4 cm	k = 0.75 (0.44–1.00)	k = 0.59 (0.08–1.00)
TS ^4^ > 4 cm	k = 0.67 (0.27–1.00)	k = 0.71 (0.34–1.00)
parametrial infiltration	k = 1.00 (1.00–1.00)	k = 0.43 (0.08–1.00)
infiltration of vaginal lower third	k = 0.81 (0.61–1.00)	k = 1.00 (1.00–1.00)
infiltration of adjacent organs	k = 1.00 (1.00–1.00)	k = 1.00 (1.00–1.00)
loco-regional LN ^5^+	k = 1.00 (1.00–1.00)	k = 1.00 (1.00–1.00)

^1^ confidence intervals; ^2^ magnetic resonance imaging; ^3^ diffusion-weighted imaging with background saturation; ^4^ tumor size; ^5^ lymph node positivity.

## Data Availability

Data are available from the authors on request.

## References

[1] Wagner-Larsen K.S., Lura N., Salvesen Ø., Halle M.K., Forsse D., Trovik J., Smit N., Krakstad C., Haldorsen I.S. (2022). Interobserver agreement and prognostic impact for MRI–based 2018 FIGO staging parameters in uterine cervical cancer. Eur. Radiol..

[2] Sung H., Ferlay J., Siegel R.L., Laversanne M., Soerjomataram I., Jemal A., Bray F. (2021). Global Cancer Statistics 2020: GLOBOCAN Estimates of Incidence and Mortality Worldwide for 36 Cancers in 185 Countries. CA Cancer J. Clin..

[3] Bhatla N., .Aoki D., Sharma D.N., Sankaranarayanan R. (2018). Cancer of the cervix uteri. Int. J. Gynaecol. Obstet..

[4] Wright J.D., Matsuo K., Huang Y., Tergas A.I., Hou J.Y., Khoury-Collado F., Clair C.M.S., Ananth C.V., Neugut A.I., Hershman D.L. (2019). Prognostic Performance of the 2018 International Federation of Gynecology and Obstetrics Cervical Cancer Staging Guidelines. Obstet. Gynecol..

[5] de Gregorio A., Widschwendter P., Ebner F., Friedl T.W.P., Huober J., Janni W., de Gregorio N. (2020). Influence of the New FIGO Classification for Cervical Cancer on Patient Survival: A Retrospective Analysis of 265 Histologically Confirmed Cases with FIGO Stages IA to IIB. Oncology.

[6] Matsuo K., Machida H., Mandelbaum R.S., Konishi I., Mikami M. (2019). Validation of the 2018 FIGO cervical cancer staging system. Gynecol. Oncol..

[7] Wagner A.E., Pappas L., Ghia A.J., Gaffney D.K. (2013). Impact of tumor size on survival in cancer of the cervix and validation of stage IIA1 and IIA2 subdivisions. Gynecol. Oncol..

[8] Lee J.H., Lee S.W., Kim J.R., Kim Y.S., Yoon M.S., Jeong S., Kim J.H., Lee J.Y., Eom K.Y., Jeong B.K. (2017). Tumour size, volume, and marker expression during radiation therapy can predict survival of cervical cancer patients: A multi-institutional retrospective analysis of KROG 16-01. Gynecol. Oncol..

[9] Frumovitz M., Sun C.C., Schmeler K.M., Deavers M.T., dos Reis R., Levenback C.F., Ramirez P.T. (2009). Parametrial involvement in radical hysterectomy specimens for women with early-stage cervical cancer. Obstet. Gynecol..

[10] Canaz E., Ozyurek E.S., Erdem B., Talmac M.A., Ozaydin I.Y., Akbayir O., Numanoglu C., Ulker V. (2017). Preoperatively Assessable Clinical and Pathological Risk Factors for Parametrial Involvement in Surgically Treated FIGO Stage IB-IIA Cervical Cancer. Int. J. Gynecol. Cancer.

[11] Koyama T., Tamai K., Togashi K. (2007). Staging of carcinoma of the uterine cervix and endometrium. Eur. Radiol..

[12] Jemal A., Siegel R., Ward E. (2006). Cancer statistics, 2006. CA Cancer J. Clin..

[13] Manganaro L., Lakhman Y., Bharwani N., Gui B., Gigli S., Vinci V., Rizzo S., Kido A., Cunha T.M., Sala E. (2021). Staging, recurrence and follow-up of uterine cervical cancer using MRI: Updated Guidelines of the European Society of Urogenital Radiology after revised FIGO staging 2018. Eur. Radiol..

[14] Cibula D., Pötter R., Planchamp F., Avall-Lundqvist E., Fischerova D., Haie Meder C., Köhler C., Landoni F., Lax S., Lindegaard J.C. (2018). The European Society of Gynaecological Oncology/European Society for Radiotherapy and Oncology/European Society of Pathology guidelines for the management of patients with cervical cancer. Radiother. Oncol..

[15] Thomeer M.G., Gerestein C., Spronk S., van Doorn H.C., van der Ham E., Hunink M.G. (2013). Clinical examination versus magnetic resonance imaging in the pretreatment staging of cervical carcinoma: Systematic review and meta-analysis. Eur. Radiol..

[16] Woo S., Suh C.H., Kim S.Y., Cho J.Y., Kim S.H. (2017). Magnetic resonance imaging for detection of parametrial invasion in cervical cancer: An updated systematic review and meta-analysis of the literature between 2012 and 2016. Eur. Radiol..

[17] Zhang W., Zhang J., Yang J., Xue H., Cao D., Huang H., Wu M., Cui Q., Chen J., Lang J. (2014). The role of magnetic resonance imaging in pretreatment evaluation of early-stage cervical cancer. Int. J. Gynecol. Cancer.

[18] Balcacer P., Shergill A., Litkouhi B. (2019). MRI of cervical cancer with a surgical perspective: Staging, prognostic implications and pitfalls. Abdom. Radiol..

[19] Van Vierzen P.B., Massuger L.F., Ruys S.H., Barentsz J.O. (1998). Fast dynamic contrast enhanced MR imaging of cervical carcinoma. Clin. Radiol..

[20] Becker M., Monnier Y., de Vito C. (2022). MR Imaging of Laryngeal and Hypopharyngeal Cancer. Magn. Reson. Imaging Clin. N. Am..

[21] Zhao C., Deng D., Ye W., Long L., Lu Y., Wei Y. (2021). Diffusion-weighted imaging with background body signal suppression (DWIBS) distinguishes benign lesions from malignant pulmonary solitary lesions. Am. J. Transl. Res..

[22] Takahara T., Imai Y., Yamashita T., Yasuda S., Nasu S., Van Cauteren M. (2004). Diffusion weighted whole body imaging with background body signal suppression (DWIBS): Technical improvement using free breathing, STIR and high resolution 3D display. Radiat. Med..

[23] Sun M., Cheng J., Zhang Y., Bai J., Wang F., Meng Y., Li Z. (2017). Application of DWIBS in malignant lymphoma: Correlation between ADC values and Ki-67 index. Eur. Radiol..

[24] Balaji R., Devi R., Stumpf J. (2013). Diffusion-Weighted Whole-Body Imaging with Background Body Signal Suppression (DWIBS)—Application in Planning for Cyberknife Therapy in Patients With Gliomas. Pract. Radiat. Oncol..

[25] Kumasaka S., Motegi S., Kumasaka Y., Nishikata T., Otomo M., Tsushima Y. (2022). Whole-body magnetic resonance imaging (WB-MRI) with diffusion-weighted whole-body imaging with background body signal suppression (DWIBS) in prostate cancer: Prevalence and clinical significance of incidental findings. Br. J. Radiol..

[26] Ishiguchi H., Ito S., Kato K., Sakurai Y., Kawai H., Fujita N., Abe S., Narita A., Nishio N., Muramatsu H. (2018). Diagnostic performance of 18F-FDG PET/CT and whole-body diffusion-weighted imaging with background body suppression (DWIBS) in detection of lymph node and bone metastases from pediatric neuroblastoma. Ann. Nucl. Med..

[27] Larsen S.K.A., Løgager V., Bylov C., Nellemann H., Agerbæk M., Als A.B., Pedersen E.M. (2022). Can whole-body MRI replace CT in management of metastatic testicular cancer? A prospective, non-inferiority study. J. Cancer Res. Clin. Oncol..

[28] Schicho A., Habicher W., Wendl C., Stroszczynski C., Strotzer Q., Dollinger M., Schreyer A.G., Schleder S. (2022). Clinical Value of Diffusion-Weighted Whole-Body Imaging with Background Body Signal Suppression (DWIBS) for Staging of Patients with Suspected Head and Neck Cancer. Tomography.

[29] Schleder S., May M., Habicher W., Dinkel J., Schreyer A.G., Gostian A.-O., Schicho A. (2022). Additional Diffusion-Weighted Imaging with Background Body Signal Suppression (DWIBS) Improves Pre-Therapeutical Detection of Early-Stage (pT1a) Glottic Cancer: A Feasibility and Interobserver Reliability Study. Diagnostics.

[30] Choi S.H., Kim S.H., Choi H.J., Park B.K., Lee H.J. (2004). Preoperative magnetic resonance imaging staging of uterine cervical carcinoma: Results of prospective study. J. Comput. Assist. Tomogr..

[31] Brennan P., Silman A. (1992). Statistical methods for assessing observer variability in clinical measures. BMJ.

[32] Pálsdóttir K., Fridsten S., Blomqvist L., Alagic Z., Fischerova D., Gaurilcikas A., Hasselrot K., Jäderling F., Testa A.C., Sundin A. (2021). Interobserver agreement of transvaginal ultrasound and magnetic resonance imaging in local staging of cervical cancer. Ultrasound Obstet. Gynecol..

[33] Leitlinienprogramm Onkologie (Deutsche Krebsgesellschaft, Deutsche Krebshilfe, AWMF): S3-Leitlinie Diagnostik, Therapie und Nachsorge der Patientin mit Zervixkarzinom, Langversion, 2.2, 2022, AWMF-Registernummer: 032/033OL. https://www.leitlinienprogramm-onkologie.de/leitlinien/zervixkarzinom/.

[34] Liu B., Gao S., Li S. (2017). A Comprehensive Comparison of CT, MRI, Positron Emission Tomography or Positron Emission Tomography/CT, and Diffusion Weighted Imaging-MRI for Detecting the Lymph Nodes Metastases in Patients with Cervical Cancer: A Meta-Analysis Based on 67 Studies. Gynecol. Obstet. Investig..

[35] Testa A.C., Di Legge A., De Blasis I., Moruzzi M.C., Bonatti M., Collarino A., Rufini V., Manfredi R. (2014). Imaging techniques for the evaluation of cervical cancer. Best Pract Res. Clin. Obstet. Gynaecol..

[36] Epstein E., Testa A., Gaurilcikas A., Di Legge A., Ameye L., Atstupenaite V., Valentini A.L., Gui B., Wallengren N.-O., Pudaric S. (2013). Early-stage cervical cancer: Tumor delineation by magnetic resonance imaging and ultrasound—A European multicenter trial. Gynecol. Oncol..

